# Relationship Between the COVID-19 Pandemic and Ecological, Economic, and Social Conditions

**DOI:** 10.3389/fpubh.2021.694191

**Published:** 2021-07-23

**Authors:** Attila Murányi, Bálint Varga

**Affiliations:** ^1^Independent Researcher, Budapest, Hungary; ^2^Department of Computer Science, Institute of Mathematics, Eötvös Loránd University, Budapest, Hungary

**Keywords:** infection rate, Ecological Footprint, Biological Capacity, median age, urban population, GDP per capita, ecosystem, coexistence

## Abstract

The COVID-19 pandemic had huge impacts on the global world, with both a negative impact on society and economy but a positive one on nature. But this universal effect resulted in different infection rates from country to country. We analyzed the relationship between the pandemic and ecological, economic, and social conditions. All of these data were collected in 140 countries at six time points. Correlations were studied using univariate and multivariate regression models. The world was interpreted as a single global ecosystem consisting of ecosystem units representing countries. We first studied 140 countries around the world together, and infection rates were related to per capita GDP, Ecological Footprint, median age, urban population, and Biological Capacity, globally. We then ranked the 140 countries according to infection rates. We created four groups with 35 countries each. In the first group of countries, the infection rate was very high and correlated with the Ecological Footprint (consumption) and GDP per capita (production). This group is dominated by developed countries, and their ecological conditions have proved to be particularly significant. In country groups 2, 3, and 4, infection rates were high, medium, and low, respectively, and were mainly related to median age and urban population. In the scientific discussion, we have interpreted why infection rates are very high in developed countries. Sustainable ecosystems are balanced, unlike the ecosystems of developed countries. The resilience and the health of both natural ecosystems and humans are closely linked to the world of microbial communities, the microbiomes of the biosphere. It is clear that both the economy and society need to be in harmony with nature, creating sustainable ecosystems in developed countries as well.

## Introduction

The first wave of the COVID-19 pandemic shocked humanity and severely limited the functioning of society and the economy. Many scientists believe that the COVID-19 pandemic is a stage in a process, a logical consequence of the degradation and depletion of nature. Can the COVID-19 pandemic be interpreted as a reaction of nature? We hypothesize that the characteristics of the COVID-19 pandemic are related to ecological, economic, and social conditions.

For the global analysis of a pandemic, we interpreted the world as a single global ecosystem consisting of ecosystem units representing countries. The natural and environmental conditions of the country can be described by ecological characteristics. However, a country can be described not only by ecological but also by economic and social characteristics.

The COVID-19 coronavirus is a biological agent from nature and has had a major impact on the global world (i.e., the global ecosystem), as well as individual countries (i.e., individual ecosystem units). In principle, the rate of COVID-19 coronavirus infection should be the same in all countries, but this is not the case. We hypothesized that the effect of COVID-19 in different countries would depend not only on the virus and its spread but also on ecological, economic, and social conditions. Although every country is made up of nature, the economy, and human society, nature is under pressure from our ever-increasing production and ever-increasing consumption.

## Materials and Methods

The pandemic was analyzed during the first wave. Pandemic data were downloaded from the https://www.worldometers.info/coronavirus/website at six time points (April 18, May 2, May 16, May 30, June 18, and July 4). The Worldometer provides live statistics and official real-time data on the COVID-19 pandemic from all over the world (1). The use of relative data to compare different countries was preferred, so the effect of COVID-19 coronavirus was characterized by the infection rate. The infection rate is the total number of cases per million people reported by countries.

The state of nature and the state of the environment were characterized by the ecological conditions of the country. The ecological conditions were characterized by the Ecological Footprint and Biological Capacity. The Ecological Footprint per person is defined as the area used to support the consumption of the country divided by the population. The Biological Capacity of a given country is the capacity of the ecosystem to produce biological materials used by the country and to absorb waste materials generated by the country. The Biological Capacity per person is the Biological Capacity of the country divided by the population. Their unit of measurement is the global hectares per person (2).

The economy was characterized by GDP per capita. GDP per capita is gross domestic product divided by mid-year population. GDP is the sum of gross value added by all resident producers in the economy plus any product taxes and minus any subsidies not included in the value of the products. It is calculated without making deductions for depreciation of fabricated assets or for depletion and degradation of natural resources. Data are in current U.S. dollars (3). Society was characterized by the median age of the population (year), urban population (%), population density (persons per square km), and the number of inhabitants and migrants (4). To study the global ecosystem, we were able to use data from a total of 140 countries in which all of these data were available.

The correlations between the infection rate and the ecological, economic, and social conditions were studied by univariate and multivariate regression models. In the case of multiple regression, the backward elimination process was used to select the most significant predictor set from the eight initial variables, using *p*-value of 0.05 as a stopping criterion. The fit of the model was characterized by *R*^2^, *F*-, and *P*-values from the ANOVA table. The calculations were made, using the STATISTICA software.

## Results

### Infection Rates on a Global Scale

All 140 countries were taken into account in characterizing the global ecosystem. Data from 140 countries were used at six time points.

We first used a univariate model to characterize the relationships between the infection rate and individual ecological, economic, and social conditions. The results were ranked according to *R*^2^ (Table 1). The infection rate can best be explained by GDP per capita, Ecological Footprint, median age, and urban population during the first wave of the COVID-19 epidemic. The importance of each variable has changed over time, which is also reflected in the *R*^2^ ranking. For example, as the epidemic progressed, the importance of the population living in the urban areas increased. The strongest correlation was obtained on April 18, 2020 (*R*^2^ = 0.7096) between the infection rate and GDP/capita, when the regression equation could explain 70.96 % of the infection rate. In case of GDP/capita, Ecological Footprint, median age, and urban population, *R*^2^ ranged from 0.30 to 0.71, 0.45 to 0.59, 0.24 to 0.36, and from 0.28 to 0.37, respectively. These results show the relationship between the infection rate and ecological, economic, and social characteristics.

**Table 1:** Univariate regression equations between the infection rate and the ecological, economic, social conditions on a global scale (140 countries, six time points).

**Regression equation**	***R*^**2**^**	***F* value**	***p* value**
**18-Apr-2020**
Cases/1 M = 0.0328 * GDP/capita	0.7096	340	3.86E-39
Cases/1 M = 176.2 * Ecofootprint	0.5033	141	7.18E-23
Cases/1 M = 17.71 * Median age	0.3340	70	6.20E-14
Cases/1 M = 862.8 * Urban pop.	0.3151	64	4.47E-13
**2-May-2020**
Cases/1 M = 0.0428 * GDP/capita	0.6958	318	9.76E-38
Cases/1 M = 245.2 * Ecofootprint	0.5618	178	1.11E-26
Cases/1 M = 24.00 * Median age	0.3535	76	7.70E-15
Cases/1 M = 1198 * Urban pop.	0.3501	75	1.11E-14
**16-May-2020**
Cases/1 M = 0.0532 * GDP/capita	0.6181	225	7.59E-31
Cases/1 M = 331.1 * Ecofootprint	0.5884	199	1.40E-28
Cases/1 M = 1606 * Urban pop.	0.3613	79	3.26E-15
Cases/1 M = 31.14 * Median age	0.3417	72	2.74E-14
**30-May-2020**
Cases/1 M = 436.4 * Ecofootprint	0.5334	159	8.97E-25
Cases/1 M = 0.0646 * GDP/capita	0.4749	126	3.53E-21
Cases/1M = 2096 * Urban pop.	0.3211	66	2.40E-13
Cases/1 M = 39.29 * Median age	0.2839	55	1.04E-11
**18-Jun-2020**
Cases/1 M = 593.1 * Ecofootprint	0.4589	118	2.86E-20
Cases/1 M = 0.0804 * GDP/capita	0.3432	73	2.33E-14
Cases/1 M = 2916 * Urban pop.	0.2896	57	5.88E-12
Cases/1 M = 53.08 * Median age	0.2413	44	6.22E-10
**4-Jul-2020**
Cases/1 M = 719.4 * Ecofootprint	0.4532	115	5.96E-20
Cases/1 M = 3679 * Urban pop.	0.3094	62	8.03E-13
Cases/1 M = 0.0931 * GDP/capita	0.3086	62	8.71E-13
Cases/1 M = 66.33 * Median age	0.2529	47	2.08E-10

As a second step, we analyzed the global ecosystem, using multivariate analysis (Table 2). The infection rate during the first wave of the COVID-19 epidemic can be explained by five characteristics: GDP/capita, Ecological Footprint, median age, urban population, and Biological Capacity. Four regression equations contain two or three independent variables that result in the best fit. The relationship between the infection rate and ecological, economic, and social conditions resulted in a better correlation when multivariate analysis was used. However, for 140 countries, the values of the individual characteristics vary widely, so it is not recommended to apply these regression equations to individual countries. Of course, these relationships are not causal, but they demonstrate that infection rates are also related to ecological, economic, and social conditions.

**Table 2:** Multivariate regression equations between the infection rate and ecological, economic, social conditions on a global scale (140 countries, six time points).

**Date**	**Regression equation**	***R*^**2**^**	***F* value**	***p* value**
18-Apr-2020	Cases/1 M = 0.0328 * GDP/capita	0.7096	340	3.86E-39
2-May-2020	Cases/1 M = 0.0428 * GDP/capita	0.6958	318	9.76E-38
16-May-2020	Cases/1 M = 0.0297 * GDP/capita + 275.8 * Ecofootprint – 14.93 * Median age	0.6715	93	5.91E-33
30-May-2020	Cases/1 M = 679.1 * Ecofootprint - 30.78 * Median age – 27.02 * Biocapacity	0.5919	66	1.59E-26
18-Jun-2020	Cases/1 M = 918.2 * Ecofootprint - 45.92 * Median age	0.5017	69	1.34E-21
4-Jul-2020	Cases/1 M = 969.5 * Ecofootprint + 3152 * Urban pop. – 98.58 * Median age	0.5000	46	1.61E-20

The correlations (*R*^2^) decreased as the pandemic progressed, which may be caused by a number of factors. On the one hand, we selected only eight characteristics that we considered to be the most significant. On the other hand, neither the characteristics of the spread of the epidemic nor the effects of the epidemic management were taken into account. Thus, the increasing values of (1–R^2^) might be explained by external factors that were not taken into account.

In the global study of the COVID-19 pandemic, 140 countries form a single group, which is best characterized by the averages of each characteristic. The average for 140 countries was as follows: GDP/capita = 14,647 USD/capita; Ecological Footprint = 3.2 gha/person; Biological Capacity = 3.9 gha/person; urban population = 60%, and median age = 31 years. The 140 countries studied were the same, so these averages were constant at six time points.

### The Infection Rate in Different Country Groups

We wanted to analyze in more detail the global relationship between the infection rate and ecological, economic, and social conditions. We were wondering why the infection rate varies from country to country. We ranked the 140 countries according to infection rates. We created four groups with 35 countries each. In the first group of 35 countries, the infection rate was very high. In groups 2, 3, and 4 of countries, the infection rates were high, medium, and low, respectively (Table 3). Ranking and grouping were performed at all six time points.

**Table 3:** Averages of ecological, economic, social conditions in the four country groups at six time points.

**Date**	**Country rank**	**Country group**	**Infection rate**	**Cases/1 M**	**GDP/capita USD**	**Ecological Footprint gha/pers**.	**Biological Capacity gha/pers**.	**Urban population %**	**Median age years**	**Population density pers./km^**2**^**
18-Apr-2020	1–35	First	Very high	1 567	38 871	5.6	3.1	76%	40	172
2-May-2020	1–35	First	Very high	2 149	37 394	5.5	3.3	77%	39	129
16-May-2020	1–35	First	Very high	2 797	37 610	5.4	3.2	78%	39	169
30-May-2020	1–35	First	Very high	3 601	36 786	5.5	3.1	79%	38	167
18-Jun-2020	1–35	First	Very high	4 943	33 254	5.2	3.7	78%	37	129
4-Jul-2020	1–35	First	Very high	6 101	30 531	5.1	3.7	77%	36	123
18-Apr-2020	36–70	Second	High	221	14 166	3.6	5.0	71%	37	87
2-May-2020	36–70	Second	High	315	13 788	3.5	3.7	69%	36	84
16-May-2020	36–70	Second	High	455	14 100	3.6	4.2	69%	36	86
30-May-2020	36–70	Second	High	599	13 174	3.3	4.0	65%	35	79
18-Jun-2020	36–70	Second	High	883	13 250	3.2	2.9	62%	34	155
4-Jul-2020	36–70	Second	High	1 305	15 099	3.2	5.2	63%	33	158
18-Apr-2020	71–105	Third	Medium	34	3 628	1.9	5.1	55%	24	138
2-May-2020	71–105	Third	Medium	64	5 310	2.1	3.9	55%	26	192
16-May-2020	71–105	Third	Medium	114	4 655	1.8	3.7	53%	26	149
30-May-2020	71–105	Third	Medium	173	6 259	2.1	4.0	57%	28	154
18-Jun-2020	71–105	Third	Medium	278	9 651	2.5	6.9	60%	29	108
4-Jul-2020	71–105	Third	Medium	382	10 525	2.6	4.8	59%	31	110
18-Apr-2020	106–140	Fourth	Low	4	1 922	1.7	2.3	40%	22	96
2-May-2020	106–140	Fourth	Low	7	2 108	1.7	4.7	41%	22	88
16-May-2020	106–140	Fourth	Low	16	2 186	1.7	4.4	41%	22	90
30-May-2020	106–140	Fourth	Low	27	2 332	1.8	4.3	41%	22	95
18-Jun-2020	106–140	Fourth	Low	46	2 394	1.8	1.9	43%	23	102
4-Jul-2020	106–140	Fourth	Low	68	2 384	1.8	1.8	42%	23	104

The dynamics and management of the epidemic varied from country to country, so the composition of the groups varied. The four groups of countries were characterized by averages that were also not constant over time. In the four groups of countries, not only the infection rate but also the ecological, economic, and social conditions differed significantly (Table 3).

The infection rate in group 1 is very high and ranges from 1,567 to 6,101 cases per million people. In groups 2, 3, and 4, the infection rates were 221–1,305, 34–382, and 4–68 cases/million people, respectively. The range of group 1 is significantly higher than that of the other three groups. There is no overlap between groups 1 and 2.

The GDP ranges per capita differ significantly in the four groups, so they do not overlap in either case. In groups 1, 2, 3, and 4, per capita GDP was $30,531–38,871, $13,174–15,099, $3,628–10,525, and $1,922–2,394, respectively. The higher the range of GDP per capita, the higher was the infection rate. GDP per capita characterizes production. This means that the higher infection rates have been reported in groups of countries with higher per capita GDP and higher production.

The state of nature (the state of the environment) is characterized by the Ecological Footprint and Biological Capacity. In groups 1, 2, 3, and 4 of the countries, the Ecological Footprint per capita is 5.1–5.6, 3.2–3.6, 1.8–2.6, and 1.7–1, 8 global hectares, respectively, and the ranges do not overlap. The larger the range of the Ecological Footprint, the higher was the range of the infection rate. The Ecological Footprint characterizes consumption. Higher infection rates have been reported in groups of countries with higher Ecological Footprints and higher consumption.

In groups 1, 2, 3, and 4 of the countries, the urban population declined systematically and ranged from 76–79, 62–71, 53–60, and 40–43%, respectively. Similarly, the median age decreased in groups 1, 2, 3, and 4 of the countries and ranged between 36–40, 33–37, 24–31, and 22–23 years, respectively.

The ranking of 140 countries by the infection rate resulted in four groups of countries with different ecological, economic, and social characteristics.

For this reason, we also wondered whether the relationship between the infection rate and ecological, economic, and social conditions was also different. The multivariate relationships are shown in Table 4. In the first group of countries, the very high infection rate can be explained mainly by GDP per capita and the Ecological Footprint. In groups 2, 3, and 4, the infection rates were mainly associated with social characteristics (median age and urban population). It should be emphasized that the equations in Table 4 do not show causal relationships. These regression equations show that the infection rates are also related to ecological, economic, and social conditions.

**Table 4:** Multivariate regression equations between the infection rate and ecological, economic, social conditions in the four country groups at six time points.

**Date**	**Country group**	**Infection rate**	**Regression equation**	***R*^**2**^**	***F* value**	***p* value**
18-Apr-2020	First	Very high	Cases/1 M = 0.0305 * GDP/capita – 121.5 * Biocapacity + 19.72 * Median age	0.8428	57	5.91E-13
2-May-2020	First	Very high	Cases/1 M = 0.0340 * GDP/capita + 35.34 * Median age – 152.8 * Biocapacity	0.8586	65	1.09E-13
16-May-2020	First	Very high	Cases/1 M = 490.8 * Ecofootprint	0.8085	144	9.43E-14
30-May-2020	First	Very high	Cases/1 M = 647.2 * Ecofootprint	0.7340	94	2.62E-11
18-Jun-2020	First	Very high	Cases/1 M = 1471 * Ecofootprint – 0.0843 * GDP/capita	0.7041	39	1.87E-09
4-Jul-2020	First	Very high	Cases/1M = 1744 * Ecofootprint – 0.0990 * GDP/capita	0.6930	37	3.45E-09
18-Apr-2020	Second	High	Cases/1 M = 5.957 * Median age	0.7683	113	2.46E-12
2-May-2020	Second	High	Cases/1 M = 8.817 * Median age	0.7887	127	5.07E-13
16-May-2020	Second	High	Cases/1 M = 12.45 * Median age	0.7935	131	3.42E-13
30-May-2020	Second	High	Cases/1 M = 14. 26 * Median age + 21.39 * Biocapacity	0.8454	90	4.18E-14
18-Jun-2020	Second	High	Cases/1 M = 1386 * Urban pop.	0.8933	285	4.30E-18
4-Jul-2020	Second	High	Cases/1 M = 2005 * Urban pop.	0.8956	292	2.99E-18
18-Apr-2020	Third	Medium	Cases/1 M = 34.35 * Urban pop. + 8.940 * Ecofootprint – 0.3819 * Biocapacity	0.9232	128	6.45E-18
2-May-2020	Third	Medium	Cases/1 M = 1.190 * Median age + 53.08 * Urban pop. + 0.8396 * Biocapacity	0.9066	104	1.48E-16
16-May-2020	Third	Medium	Cases/1 M = 4.076 * Median age	0.8319	168	1.01E-14
30-May-2020	Third	Medium	Cases/1 M = 5.867 * Median age	0.8432	183	3.08E-15
18-Jun-2020	Third	Medium	Cases/1 M = 8.649 * Median age	0.8062	141	1.16E-13
4-Jul-2020	Third	Medium	Cases/1 M = 11.34 * Median age	0.7774	119	1.24E-12
18-Apr-2020	Fourth	Low	Cases/1 M = 1.478 * Ecofootprint + 0.0141 * Population density	0.7164	42	9.31E-10
2-May-2020	Fourth	Low	Cases/1 M = 13.65 * Urban pop. + 0.0184 * Population density	0.6812	35	6.43E-09
16-May-2020	Fourth	Low	Cases/1 M = 6.038 * Ecofootprint + 0.0372 * Population density	0.5750	22	7.40E-07
30-May-2020	Fourth	Low	Cases/1 M = 59.03 * Urban pop.	0.6166	55	1.42E-08
18-Jun-2020	Fourth	Low	Cases/1 M = 99.73 * Urban pop.	0.7093	83	1.21E-10
4-Jul-2020	Fourth	Low	Cases/1 M = 149.4 * Urban pop.	0.7403	97	1.73E-11

When the global world was analyzed as a single ecosystem, the regression equations were able to explain 50–70% of the infection rate (Table 2). However, categorization of countries by the infection rate resulted in a closer correlation with *R*^2^ growth (Table 4). In the four groups of countries, the selected variables explain 69–86, 76–90, 77–93, and 57–75% of the infection rate, respectively. The regression equations of four groups of countries better describe the relationship between the infection rate and ecological, economic, and social conditions.

### The Country Group With a Very High Infection Rate

We found that the four country groups have different ecological, economic, and social characteristics. However, the first group deserves special attention due to the very high infection rate as well as the highest per capita GDP and Ecological Footprint (Table 3). This group is dominated by developed countries with the highest production (GDP/capita) and consumption (Ecological Footprint/person). But why was the infection rate highest in the developed group of countries?

High production and high consumption obviously cannot cause infection. In developed countries, however, high industrial and agricultural production and high consumption have long had a direct and indirect impact on nature. In other words, nature has long been under great pressure in developed countries. As a result of the cumulative effects, the ecological characteristics of the ecosystem may have changed. This is supported by the data in Table 3. The Ecological Footprint is significantly higher in the first group of countries (5.6–5.1) than in the other three groups of countries (3.6–1.7). In addition, the ecosystem is unbalanced because the Ecological Footprint is always much higher than the Biological Capacity. This is not the case in the other three groups of countries, where the Ecological Footprint is, in most cases, lower than the Biological Capacity. So, the importance of ecological characteristics is not negligible, especially in developed countries.

For this reason, we studied the ecological characteristics of country group 1 in more detail. We can analyze the ecological characteristics of a total of 46 countries (Table 5). The countries are ranked according to infection rates. The Ecological Footprint exceeds four in 32 countries. The Ecological Footprint is highest (>8) in Luxemburg, Qatar, United Arab Emirates, and the United States. Biological capacity <1 in 10 countries where the ecosystem is not able to produce enough biological material for the population. The ecosystem of a country is unbalanced if its Ecological Footprint is much larger than its Biological Capacity. This occurs in 11 countries where the infection rate is also very high. The biocapacity deficit (i.e., overconsumption) is very high (< -4) in Luxembourg, Qatar, the United Arab Emirates, Belgium, the United Kingdom, the United States, Israel, the Netherlands, Saudi Arabia, Oman, and Malta. Excessive consumption in these countries results in ecosystem imbalances.

**Table 5:** Ecological characteristics in countries with very high infection rates (country group 1, six time points).

**18-Apr-2020**	**Cases/1 M**	**Ecological Footprint gha/per**.	**Biological Capacity gha/pers**.	**2-May-2020**	**Cases/1 M**	**Ecological Footprint gha/pers**.	**Biological Capacity gha/pers**.	**16-May-2020**	**Cases/1 M**	**Ecological Footprint gha/pers**.	**Biological Capacity gha/pers**.
Luxemb.	5 650	15.3	1.8	Luxemb.	6 074	15.3	1.8	Qatar	10 774	14.4	1.0
Spain	4 158	4.0	1.4	Spain	5 252	4.0	1.4	Luxemb.	6 280	15.3	1.8
Belgium	3 322	6.3	0.8	Qatar	5 162	14.4	1.0	Spain	5 914	4.0	1.4
Switzerland	3 166	4.6	1.0	Belgium	4 273	6.3	0.8	Ireland	4 859	5.1	3.4
Ireland	2 989	5.1	3.4	Ireland	4 219	5.1	3.4	Belgium	4 747	6.3	0.8
Italy	2 910	4.4	0.9	Switzerland	3 445	4.6	1.0	USA	4 498	8.1	3.6
France	2 325	4.5	2.4	Italy	3 431	4.4	0.9	Italy	3 702	4.4	0.9
USA	2 232	8.1	3.6	USA	3 420	8.1	3.6	UK	3 540	6.6	1.1
Portugal	1 931	4.1	1.3	UK	2 614	6.6	1.1	Switzerland	3 536	4.6	1.0
Netherlands	1 844	4.9	0.8	France	2 564	4.5	2.4	Belarus	3 035	4.6	3.4
Qatar	1 738	14.4	1.0	Portugal	2 486	4.1	1.3	Sweden	2 941	6.4	9.6
Germany	1 715	4.9	1.6	Netherlands	2 348	4.9	0.8	Portugal	2 824	4.1	1.3
UK	1 682	6.6	1.1	Sweden	2 186	6.4	9.6	France	2 751	4.5	2.4
Israel	1 544	4.9	0.3	Germany	1 958	4.9	1.6	Netherlands	2 561	4.9	0.8
Sweden	1 369	6.4	9.6	Israel	1 866	4.9	0.3	UAE	2 211	8.9	0.6
Panama	990	2.3	2.8	Belarus	1 675	4.6	3.4	Panama	2 152	2.3	2.8
Turkey	976	3.4	1.5	Panama	1 557	2.3	2.8	Germany	2 099	4.9	1.6
Canada	885	7.7	15.1	Ecuador	1 493	1.8	2.0	Chile	2 071	4.3	3.5
UAE	686	8.9	0.6	Canada	1 459	7.7	15.1	Canada	1 979	7.7	15.1
Moldova	589	1.7	1.2	Turkey	1 451	3.4	1.5	Israel	1 922	4.9	0.3
Ecuador	511	1.8	2.0	UAE	1 318	8.9	0.6	Ecuador	1 787	1.8	2.0
Chile	509	4.3	3.5	Moldova	987	1.7	1.2	Turkey	1 739	3.4	1.5
Belarus	506	4.6	3.4	Chile	890	4.3	3.5	Moldova	1 424	1.7	1.2
**30-May-2020**	**Cases/ 1M**	**Ecological Footprint gha/pers**.	**Biological Capacity gha/pers**.	**18-Jun-2020**	**Cases/ 1M**	**Ecological Footprint gha/pers**.	**Biological Capacity gha/pers**.	**4-Jul-2020**	**Cases/ 1M**	**Ecological Footprint gha/pers**.	**Biological Capacity gha/pers**.
Qatar	19 211	14.4	1.0	Qatar	30 074	14.4	1.0	Qatar	35 324	14.4	1.0
Luxemb.	6 425	15.3	1.8	Chile	11 779	4.3	3.5	Chile	15 070	4.3	3.5
Spain	6 124	4.0	1.4	USA	6 791	8.1	3.6	USA	8 833	8.1	3.6
USA	5 459	8.1	3.6	Luxemb.	6 540	15.3	1.8	Panama	8 342	2.3	2.8
Ireland	5 054	5.1	3.4	Spain	6 253	4.0	1.4	Luxemb.	7 150	15.3	1.8
Belgium	5 022	6.3	0.8	Belarus	5 996	4.6	3.4	Sweden	7 071	6.4	9.6
Chile	4 966	4.3	3.5	Sweden	5 550	6.4	9.6	Belarus	6 696	4.6	3.4
Belarus	4 408	4.6	3.4	Panama	5 240	2.3	2.8	Spain	6 366	4.0	1.4
UK	4 021	6.6	1.1	Belgium	5 208	6.3	0.8	Belgium	5 335	6.3	0.8
Italy	3 848	4.4	0.9	Ireland	5 137	5.1	3.4	Ireland	5 166	5.1	3.4
Sweden	3 677	6.4	9.6	UK	4 427	6.6	1.1	UAE	5 142	8.9	0.6
Switzerland	3 566	4.6	1.0	UAE	4 426	8.9	0.6	Moldova	4 381	1.7	1.2
UAE	3 431	8.9	0.6	Italy	3 939	4.4	0.9	Portugal	4 273	4.1	1.3
Portugal	3 157	4.1	1.3	Portugal	3 735	4.1	1.3	UK	4 197	6.6	1.1
Panama	2 908	2.3	2.8	Switzerland	3 606	4.6	1.0	Italy	3 993	4.4	0.9
France	2 863	4.5	2.4	Moldova	3 249	1.7	1.2	Switzerland	3 720	4.6	1.0
Netherlands	2 700	4.9	0.8	Netherlands	2 878	4.9	0.8	Ecuador	3 438	1.8	2.0
Canada	2 391	7.7	15.1	Ecuador	2 785	1.8	2.0	Israel	3 156	4.9	0.3
Ecuador	2 189	1.8	2.0	Canada	2 654	7.7	15.1	Netherlands	2 938	4.9	0.8
Germany	2 187	4.9	1.6	France	2 431	4.5	2.4	Canada	2 787	7.7	15.1
Moldova	2 007	1.7	1.2	Germany	2 263	4.9	1.6	France	2 558	4.5	2.4
Turkey	1 936	3.4	1.5	Turkey	2 183	3.4	1.5	Turkey	2 426	3.4	1.5
Israel	1 850	4.9	0.3	Israel	2 163	4.9	0.3	Germany	2 354	4.9	1.6

In 23 countries, the infection rate was very high at all six time points. Of these 23 countries, 14 belong to Europe, four to Asia (Middle East), three to North America, and two to South America. This group of countries is dominated by developed countries, and their ecological characteristics have proved to be particularly significant. In these 23 countries (where infection rates have always been very high), either the Ecological Footprint is high, or the Biological Capacity is low, or the ecosystem is unbalanced.

**Figure 1:**
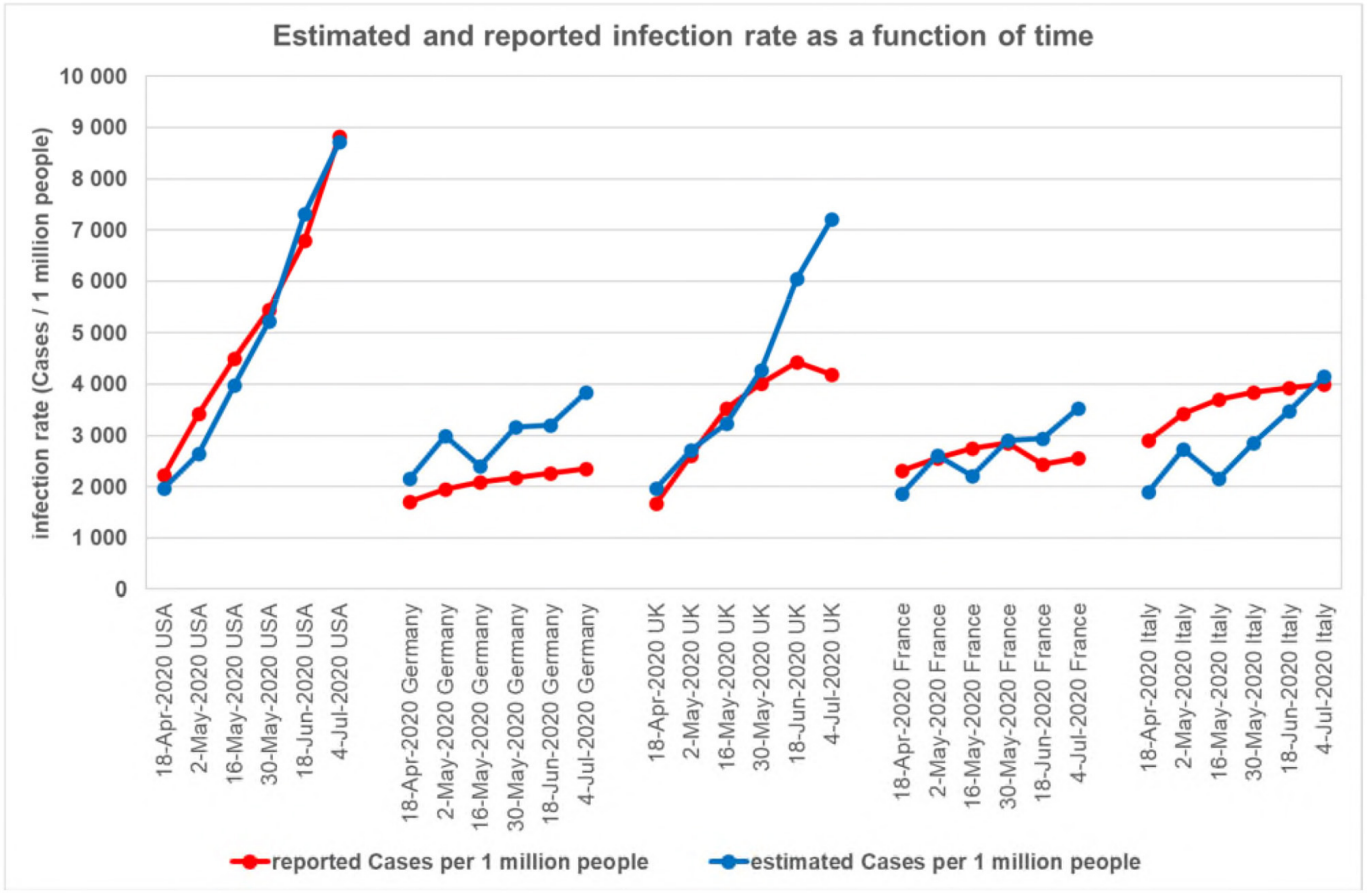
Reported and estimated infection rates at six time points. X-axis: country and six time points. Y-axis: the reported and estimated infection rate (cases per one million people).

Table 5 is dominated by developed countries. The gross domestic product of the United States, Germany, the United Kingdom, France, and Italy is high and among the top 10 in the world. Their estimated and reported infection rates as a function of time are compared in Figure 1. For the estimation, we used the regression equations of the first group of countries (Table 4) as well as the ecological, economic, and social conditions of the given country.

The United States is the strongest economy in the world. The reported rate of infections per million people increased from 2,232 to 8,833, covering a wide range. The estimated and reported infection rates fit well. This indicates that the rate of infection in the strongest economy in the world is not independent of ecological, economic, and social conditions. In Germany, the reported infection rate is increasing slowly and gradually, from 1,715 to 2,354 cases/million people. In addition, the estimates resulted in higher infection rates than the reported infection rates. These may indicate effective management of the epidemic. In the UK, the reported infection rate initially increased rapidly, and the estimated and reported infection rates correlated well. It is likely that the appropriate restrictions then stabilized the rate of infection. In France, the number of reported infections was <2,600 cases per million people. Estimated and reported infection rates were two times correlated, two times underestimated, and two times overestimated. In Italy, the infection rate was high on April 18, 2020 (2,910 cases per million people were reported), and the reported infection rate was higher than the estimated infection rate. Subsequently, management of the epidemic gradually stabilized the rate of infection and reported and estimated infection rates approached each other.

To the best of our knowledge, the relationship between the COVID-19 pandemic and ecological, economic, and social conditions has not yet been studied. Our results show that country group 1 (dominated by the developed countries) represents unbalanced ecosystems. As a result, it is worth discussing the state of our ecological environment, the state of nature, with special regard to the sustainability of ecosystems.

## Discussion

### Sustainability of Ecosystems

Sustainable natural ecosystems (terrestrial ecosystems and aquatic ecosystems) are based on the ecological pyramid formed during evolution, where production and consumption are in balance.

But our global ecosystem is threatened by the impact of human activity on Earth. Steffen et al. studied the boundaries of our planet, the levels of man-made perturbations beyond which the functioning of the Earth can change significantly. According to their calculations, changes in genetic diversity and biochemical flux pose a high risk, while land use and climate change pose an increased risk to Earth stability (5). These processes can cause imbalances in the global ecosystem. In 2020, the Intergovernmental Platform on Biodiversity and Ecosystem Services (IPBES) also proposed the reassessment of the relationship between man and nature, as ecological disturbances and unsustainable consumption result in biodiversity loss, climate change, and epidemic risk (6). These conclusions demonstrate that our global ecosystem is unsustainable in the long run.

The ecosystem of a country suffers not only from global processes but also from local environmental impacts. The ecosystems of countries are at risk, especially in developed countries, where humans have a very significant impact on the natural ecosystem, its natural components, the atmosphere, water, soil, and living organisms. Unfortunately, these adverse effects are usually recognized too late (acidification, air, water, soil pollution, etc.) (7, 8).

Impacts on the environment have ecological consequences because nature is a logically functioning ecological system that responds to external influences. The resistance of natural components to adverse effects follows the order of buffer capacity: air < water < soil < living organism. Air is the most sensitive to pollution because its buffer capacity is low. However, these adverse effects have not only ecological but also economic consequences. In the U.S. economy, for example, air pollution has become less intense. As a result, gross economic damage (due to premature death) decreased (9). That is, air pollution has caused economic losses. The ecological consequences, therefore, had direct economic consequences. The economic consequences of COVID-19 can be assessed in light of the above. The pandemic can be understood as a reaction of nature. Based on this, the loss of GDP caused by the COVID-19 pandemic can be interpreted as a tax reimbursed to nature.

Urban ecosystems represent man-made ecosystems (10). The urban population lives in the urban ecosystem. It is worth noting that the characteristics of urban ecosystems and the first group of countries are similar: per capita GDP (economic production) is high, the Ecological Footprint (human consumption) is high, Biological Capacity is low, and the ecosystem is unbalanced. In the urban ecosystem, regardless of ecological characteristics, the infection can spread much more easily due to high population density, small social distance, and a large number of human contacts. These direct reasons explain why the rate of COVID-19 coronavirus infection may be related to the urban population.

In our analysis, the urban population represents the urban ecosystem. Our regression equations in Table 4 often include the urban population. For example, in country group 4, the urban population is included four times in the regression equation. In these 35 countries, the average urban population is <43% (Table 3), so the majority of people live in rural areas where the infection cannot spread easily, so the infection rate is also low. Our results suggest that the role of the urban population in the epidemic was significant, especially in countries with low urbanization.

Although the Ecological Footprint and Biological Capacity of urban ecosystems are unknown, their ecological characteristics deserve more attention. Flies et al. (11) showed that urbanization reduces the abundance and diversity of airborne microbes and suggested studying the impact of aerobiome on human health in urban ecosystems. Robinson et al. (12) studied the aerobiome of urban green spaces and found significant vertical stratification in the potentially pathogenic and beneficial bacterial taxa. It is worth noting that microorganisms living in the biosphere (as well as viruses, including pathogens) live in the same environment as we do (13).

### Microbiome in Ecosystems and Humans

Sustainable natural ecosystems, their ecological pyramids, and their evolution are based on the invisible microscopic world of microorganisms.

Microorganisms in nature are studied as microbiomes. The definition of microbiome was updated in 2020. A “microbiome” is a characteristic microbial community that occupies a fairly well-defined habitat with distinct physicochemical properties. The microbiome represents the microbiota (the community of microorganisms) as well as the “activity theater,” which includes microbial structural elements (including mobile genetic elements, such as viruses, etc.), microbial metabolites, and habitat environmental conditions (14).

Microbiomes play a vital role in both ecosystems and humans. The understanding of microbiomes in soils, plants, animals, and humans might play a key role in solving new challenges, which are associated with anthropogenic-driven changes in the field of planetary health (14). In this way, the resilience of nature to adverse effects depends on the function, sensitivity, and stability of the microbiome of the ecosystem as well. So, microbiomes play a significant role in the global ecosystem. For example, microbial motors drive the biogeochemical cycles of the Earth (15), and microbial activity mediates the fluxes of greenhouse gases (16). Recognizing the global role of microbiomes, we direct our attention to the interaction between microorganisms and the components of nature.

**Coexisting microorganisms in the soil** form the basis of the life of healthy terrestrial ecosystems (13, 17). Soil life is fundamental to the ecological pyramid of terrestrial ecosystems. In stressed ecosystems, the ability of the soil to recover is the key to maintain soil health (18). Billions of microorganisms in soil form a complex community in which hundreds of microbial species coexist. The community of soil micro-organisms (soil microbiota) lives in harmony with its surrounding environment. The long-term quality of soil is determined by its physical, chemical, and biological properties (19). Soil habitats probably contain the greatest microbial diversity on Earth (20). Soil archaea, bacteria, fungi, and viruses are globally as well as locally diverse (21). Harmful impacts on soil microbiota affect the biodiversity of soil flora and fauna, as related to soil and plant health (22). Soil fertility is greatly determined by the microbiological activity of the root environment in both healthy (23) and in polluted soil (24). Microbial diversity is critical to maintaining the multi-functionality of terrestrial ecosystems (25). Although soil vitality is dominated by microorganisms, their coexistence with soil fauna is not negligible either.

**Coexisting microorganisms with animals** influence animal health. Biodiversity in agricultural landscapes affects the protective microbiome of insects (26). The insect—microbiome interaction influences the host and the microbial symbiont (27). The microbiome associated with insects is very complex and behaves as a mini ecosystem (28), which is highly dependent on the environment (29). The biodiversity of the host-associated microbiota is recognized as an essential component of wildlife management, which has a profound impact on animal health, but these microbial communities can be drastically altered by anthropogenic activities (30). Unfortunately, the reality is that insects have declined by 40% in recent decades, and one-third are at risk (31). Global threats to insects are caused by a number of factors (agricultural intensification, insecticides, pollution, deforestation, urbanization, etc.) (32). Pesticide exposure, infectious disease, and nutritional stress contribute to honey bee mortality and a high rate of colony loss. Symbionts may be major regulators of stress tolerance and disease resistance. Missing microbes in bees, as well as systematic depletion of key symbionts, impair bee immunity. Treatment strategies based on microbiota restoration are promising in restoring bee colony health (33). Healthy microbiomes represent the foundations of soil, plant, and animal life in sustainable ecosystems. However, this is true not only for the components of the ecosystem but also for healthy people.

**Coexisting microbes with humans** support human health. Both people and society are seen as part of the ecosystem unit of a country. Human and ecosystem health are not independent of each other. Relman (34) adopted ecological perspectives to understand the health functions of the human microbiota and the resilience of the human microbial ecosystem. The number of microbes (which live inside and outside our body) is about 10 times greater than the number of our body cells (35). Therefore, the human microbiome has become the subject of intensive research to clarify its role in health and disease. For example, the skin acts as a physical barrier, preventing the invasion of foreign pathogens while providing a home to the commensal microbiota (36). Pulmonary immunity is shaped by interaction with the microbiota (37). Communication disorders between the innate immune system and the intestinal microbiota may contribute to the development of complex diseases (38).

The microbiota plays an essential role in the functioning of the host immune system, not only in the case of animals but also in the case of humans. However, in high-income countries, overuse of antibiotics, dietary changes, and so on have resulted in the selection of a microbiota that lacks the resilience and diversity needed to establish balanced immune responses (39). Our diet influences the gut microbiome and the immune system (40). The Western diet activates the innate immune system and impairs adaptive immunity, leading to chronic inflammation and impaired defense against viruses. Wider access to healthy food should be a top priority (41). (However, healthy plants can be grown in healthy soil that is full of life.) Age-related changes in the intestinal microbiota were associated with the immune system in old age (42). The immune system deteriorates with age and causes about 90% of excessive deaths in people over the age of 65 during a regular influenza season (43).

Our results are consistent with the scientific literature, because COVID-19 coronavirus infection can often be explained by median age (Table 4). For example, in groups 3 and 4, the median age was <31 years and the infection rate was medium or low (Table 3). The younger the population, the stronger the immune system and the lower the infection rate. Assuming that humans are seen as part of the natural ecosystem, it is not surprising that the resilience of both natural ecosystems and humans is closely related to the world of a healthy microbial community.

## The Future

The outbreak of the COVID-19 pandemic is a serious warning to humanity, which can choose from three main directions of development. The world of the future can be economy driven (production based) or society driven (consumption based) or nature driven (coexistence based). This can be deduced from our results and is supported by the recent scientific literature. Because COVID-19 infection is very high in developed countries, the coronavirus epidemic indicates to humanity that the only chance of survival is to live in a coexisting world. Both the economy and society must be in harmony with nature, creating sustainable ecosystems in developed countries as well.

This makes not only scientific but also economic sense. In 2020, the World Economic Forum assessed global risks in terms of probability and impact. It has been recognized that risks related to nature are underestimated in business decision-making, and the new nature economy needs to take into account the economic value of nature. According to the World Economic Forum, business rationality lies in the preservation or restoration of natural ecosystems (31).

## Data Availability Statement

Publicly available datasets were analyzed in this study. This data can be found here: https://www.worldometers.info/coronavirus/.

## Author Contributions

AM: development of the concept, data collection, data evaluation, scientific discussion, and writing. AM and BV: methodology, data analysis. All authors contributed to the article and approved the submitted version.

## Conflict of Interest

The authors declare that the research was conducted in the absence of any commercial or financial relationships that could be construed as a potential conflict of interest.

## Publisher's Note

All claims expressed in this article are solely those of the authors and do not necessarily represent those of their affiliated organizations, or those of the publisher, the editors and the reviewers. Any product that may be evaluated in this article, or claim that may be made by its manufacturer, is not guaranteed or endorsed by the publisher.
